# FDA Approvals and Consensus Guidelines for Botulinum Toxins in the Treatment of Dystonia

**DOI:** 10.3390/toxins12050332

**Published:** 2020-05-17

**Authors:** Lauren L. Spiegel, Jill L. Ostrem, Ian O. Bledsoe

**Affiliations:** Movement Disorders and Neuromodulation Center, Department of Neurology, University of California, San Francisco, CA 94115, USA; lauren.spiegel@ucsf.edu (L.L.S.); Jill.Ostrem@ucsf.edu (J.L.O.)

**Keywords:** botulinum toxin, dystonia, cervical dystonia, blepharospasm, FDA

## Abstract

In 2016, the American Academy of Neurology (AAN) published practice guidelines for botulinum toxin (BoNT) in the treatment of blepharospasm, cervical dystonia, adult spasticity, and headache. This article, focusing on dystonia, provides context for these guidelines through literature review. Studies that led to Food and Drug Administration (FDA) approval of each toxin for dystonia indications are reviewed, in addition to several studies highlighted by the AAN guidelines. The AAN guidelines for the use of BoNT in dystonia are compared with those of the European Federation of the Neurological Societies (EFNS), and common off-label uses for BoNT in dystonia are discussed. Toxins not currently FDA-approved for the treatment of dystonia are additionally reviewed. In the future, additional toxins may become FDA-approved for the treatment of dystonia given expanding research in this area.

## 1. Introduction

Commercially available botulinum toxin (BoNT) formulations in the United States include OnabotulinumtoxinA (OnaBoNT-A), AbobotulinumtoxinA (AboBoNT-A), IncobotulinumtoxinA (IncoBoNT-A) and RimabotulinumtoxinB (RimaBoNT-B). This paper discusses the Food and Drug Administration (FDA) indications of these different formulations in the treatment of cervical dystonia and blepharospasm and includes a brief review of the studies on which FDA approval were based. Established clinical guidelines by both the American Academy of Neurology (AAN) and European Federation of the Neurological Societies (EFNS) are summarized. Finally, we provide a brief review of published data for common off-label uses of BoNT for dystonia, and discuss new toxins currently in development.

## 2. Cervical Dystonia

All four commercially available botulinum toxin formulations in the US are FDA approved for use in cervical dystonia (Table 1 and Table 2). The FDA evaluated both pivotal trials and unpublished trials in its review of submitted applications for each BoNT. OnaBoNT-A was approved by the FDA for cervical dystonia in 1999, RimaBoNT-B in 2000, AboBoNT-A in 2009, and IncoBoNT-A in 2010.

### 2.1. OnabotulinumtoxinA (Botox^®^)

OnaBoNT-A was originally called “Oculinum^®^” and was first injected into humans in 1968 by an ophthalmologist, Dr. Alan Scott, to treat strabismus [7,8]. Oculinum^®^ was manufactured by Oculinum, Inc. and distributed by Allergan, Inc in the late 1970s. Allergan Inc. acquired Oculinum, Inc. in 1991 and changed the brand name to Botox^®^ [8]. Allergan conducted a multicenter, randomized, double-blind, placebo-controlled phase III study for the evaluation of OnaBoNT-A in cervical dystonia [1]. The study was divided into two periods, the first a ten-week open-label active treatment run-in period, and the second a ten-week double-blind placebo-controlled phase. The primary outcome variables of the study were the Cervical Dystonia Severity Scale (CDSS) and the physician Global Assessment Scale (GAS) six weeks after injection in period 2. In the first period, participants with cervical dystonia between ages 21 and 75 were included only if they had a baseline score of 4 or greater on the CDSS. In addition, they were required to have had prior OnaBoNT-A treatment with at least two consecutive injections of no more than 360 units (U) each. Only those demonstrating improvement in the physician GAS during period 1 were eligible for period 2. Period 2 was initiated for each qualifying participant up to six weeks after completing period 1, and only once CDSS score returned to 4 or higher. Those who did not have return of CDSS to 4 or greater within this interval were excluded from period 2.

Of 214 participants enrolled in phase 1, 18 were excluded from advancement to phase 2 because of lack of improvement in physician GAS, 2 because CDSS did not return to at least 4, and 24 for other reasons (missed visits and protocol violations). Amongst these patients, 170 advanced to phase 2, with 88 randomized to OnaBoNT-A and 82 to placebo. A total of 35 of these randomized participants did not complete phase 2. Twenty-seven withdrew from the study because they subjectively felt treatment was not effective (8 in the OnaBoNT-A group, and 19 in the placebo group), and eight were excluded due to missed visits or protocol violations.

Mean baseline CDSS scores at the beginning of period 2 were 9.2 in the treatment group and 9.3 in the placebo group. The doses and muscles targeted were determined by the injecting physician. OnaBoNT-A administration significantly improved CDSS scores at six weeks after injection by a mean of 1.81 points, compared with a mean of 0.31 points for placebo (*p* = 0.012). A greater percentage of participants showed improvement on the physician GAS at week six when treated with OnaBoNT-A (61.7%) versus placebo (41.6%) (*p* = 0.022). Botox^®^ was approved for use in cervical dystonia (CD) in 1999. The pivotal phase III study was published in 2012 [1]. The maximum dose recommended according to the FDA-label is 50 U per injection site with a total dose of 400 U for adult patients [9].

### 2.2. RimabotulinumtoxinB (Myobloc^®^)

RimaBoNT-B was approved for cervical dystonia in 2000 based on two multicenter, double-blind, placebo-controlled trials [2,3]. Injecting investigators selected two to four muscles for each participant, and all had at least two of the following injected: splenius capitis, sternocleidomastoid, levator scapulae, trapezius, semispinalis capitis, and scalene. Trials for FDA approval used a maximum of 10,000 U of RimaBoNT-B. Recommended initial dosage for cervical dystonia is 2500 to 5000 U divided among affected muscles [10]. The FDA label recommends against calculating doses of RimaBoNT-B on the basis of conversion from other toxin formulation dosing.

The first pivotal trial for RimaBoNT-B in cervical dystonia enrolled patients with a previous acceptable response to type A toxin. One hundred twenty-two predominantly Caucasian patients were randomized to receive placebo, 2500, 5000 or 10,000-unit targeted injections of RimaBoNT-B. Patients improved by 3.3 points, 11.6 points, 12.5 points, and 16.4 points respectively on TWSTRS (Toronto Western Spasmodic Torticollis Rating Scale) total score at week 4 (*p* < 0.001 for each dose compared to placebo). The study was not designed to examine efficacy duration [2]. The second study enrolled 77 patients who previously had a clinically meaningful response to type A toxin but failed to respond to the last two adequate treatment sessions or had failed one session and had positive Type A neutralizing antibodies. All participants were confirmed to have treatment nonresponse to Type A toxin on the basis of nonresponse to the frontalis test. Participants were randomized to injection with either placebo or 10,000 units of RimaBoNT-B. TWSTRS total scores at baseline were 51.2 (placebo) and 52.8 (treatment). The TWSTRS total score was reduced by 2 points (3.9%) in the placebo group and 11.1 points (21%) in the 10,000 U group (*p* = 0.012) at week 4. Primary side effects from both pivotal trials included dry mouth and mild dysphagia [3].

### 2.3. AbotulinumtoxinA (Dysport^®^)

AboBoNT-A was initially approved for cervical dystonia in 2009 at starting doses between 250 and 500 units based on two pivotal trials with injections into 2–4 clinically indicated muscles selected by the injector [4,5]. Studies submitted for FDA approval did not test doses above 1000 U. As with RimaBoNT-B, the FDA label does not recommend calculating AboBoNT-A doses on the basis of conversion from other toxin formulations [11].

Both pivotal trials were randomized, double-blind, placebo-controlled, single-dose, parallel-group studies, though the second included an open-label extension phase with variable dosing allowed. The first study, published in 2005, included 80 patients randomized to receive placebo or AboBoNT-A 500 U injections [4]. The primary outcome was change in TWSTRS total score at week 4 in comparison to baseline. Baseline TWSTRS total scores were 46.2 in the placebo group and 45.1 in the AboBoNT-A group. Evaluation at week 4 showed a 10-point (22.2%) reduction in the AboBoNT-A group, compared to a 3.8-point (8.2%) reduction in the placebo group (*p* ≤ 0.013). Blurred vision and weakness occurred significantly more often with AboBoNT-A than placebo. The AboBoNT-A median efficacy duration was 18.5 weeks [4].

A second study included a larger number of patients and also aimed to evaluate long-term safety and efficacy of repeated injections [5]. One hundred sixteen patients were randomized to receive placebo or AboBoNT-A 500 U injections in the initial double-blind, placebo-controlled phase lasting 12 weeks. An open-label extension phase followed in which investigators could increase or decrease the dose by 250 U at their discretion and based on patient response. The primary outcome measures were change in TWSTRS total score at week 4 when compared to baseline (as part of the double-blind phase) as well as after each treatment cycle in the open-label extension phase. At week 4 in the double-blind phase, the AboBoNT-A group showed a 15.6-point (35.6%) TWSTRS total score reduction compared to a 6.7-point (14.6%) reduction in the placebo group (*p* < 0.001). Improvements compared to baseline were sustained at week 12 with a 9.1-point (21%) reduction in the AboBoNT-A and 4.9-point (10.7%) reduction in the placebo groups (*p* < 0.001). In the open-label extension phase, similar reductions in TWSTRS total scores from baseline were seen. Dysphagia was the most common adverse event associated with AboBoNT-A, occurring in 9% in the treatment group in the double-blind phase and in 6–13% of patients in the open-label phase.

### 2.4. IncobotulinumtoxinA (Xeomin^®^)

In 2010, the FDA approved Xeomin^®^ for treatment of CD on the basis of a pivotal trial [6,12]. The muscles injected were at the discretion of the treating physician. Recommended initial dose on the FDA label is 120 U and maximum dose 400 U [12]. The trial was a prospective, multicenter, double-blind, randomized, placebo-controlled study in which 233 patients were randomized to receive placebo or IncoBoNT-A 120 or 240 units [6]. The baseline TWSTRS total score was 41.8 in the placebo group, 42.6 in the 120 U group, and 42.1 in the 240 U group. At week 4, the TWSTRS total score was reduced by a mean of 2.2 (5.3%), 7.5 (17.6%), and 9 points (21.4%), respectively (240 U vs. placebo *p* < 0.001 and 120 U vs. placebo *p* < 0.001). While the study was not powered to detect a difference between the two active treatment groups, there was a trend for larger mean change in TWSTRS total score in the 240 U group. Primary side effects of the treatment arm included dysphagia, muscle weakness, and neck pain [6].

## 3. Blepharospasm

OnaBoNT-A and IncoBoNT-A are FDA-approved for the treatment of blepharospasm associated with dystonia in patients 12 years of age or older. OnaBoNT-A was approved for blepharospasm in 1989 based primarily on the strong response noted in open-label observational series [13]. The basis for FDA approval also included an open label study in 27 patients [14] and a small, double-blind, placebo-controlled trial including twelve patients with blepharospasm [15]. This latter trial had a crossover design in which patients were randomized to OnaBoNT-A or placebo but would cross to the alternate treatment in double-blind fashion if no response was noted on two consecutive injections. Four patients received placebo, of whom three subsequently received OnaBoNT-A, and eight received OnaBoNT-A only. OnaBoNT-A injections resulted in significantly greater benefit than placebo (*p* < 0.005), leading to 71.6% improvement in the Fahn blepharospasm rating scale (FBRS) (*p* < 0.01), 60.7% improvement in a self-assessment score (*p* < 0.01), and 38.9% improvement in a videotaped rating score (*p* < 0.04) compared to baseline. Complications included blurring of vision in six, tearing in five, ecchymosis in four, ptosis in two, and diplopia in one [15].

The FDA initially approved IncoBoNT-A for blepharospasm in patients previously treated with OnaBoNT-A. Approval was based on a multicenter, randomized, double-blind, placebo-controlled phase III trial, in which 75 patients were administered up to 50 U of IncoBoNT-A per eye and 34 patients were administered placebo injections [16]. All patients except one had previously experienced satisfactory benefit from two prior treatments with OnaBoNT-A. The primary outcome variable was the week 6 change from baseline in the Jankovic rating scale (JRS) severity subscore. The mean dose administered was 66.9 U with a large range from 20 to 100 U. At 6 weeks, the JRS severity subscore was reduced in the treatment arm by 0.83 points and increased in the placebo arm by 0.21 points, resulting in a statistically significant difference (*p* < 0.001). Xeomin^®^ was approved for blepharospasm in 2010 [12].

The FDA label for IncoBoNT-A was expanded to include treatment of BoNT-naïve patients in 2019 based on additional data from a multicenter, randomized, double-blind, placebo-controlled trial with results presented at the American Academy of Neurology 2019 Annual Meeting (not yet published) [12,17]. In this trial, 61 patients with a JRS severity subscore of at least two were randomized to placebo or IncoBoNT-A, and 55 patients finished the placebo-controlled phase. Twenty patients were randomized to placebo, 22 to a 25 U (12.5 U per eye) treatment group, and 19 patients to a 50 U (25 U per eye) treatment group. The 50 U treatment group was reported to demonstrate statistically significant improvements compared to placebo, with a difference of −1.2 points on the JRS severity subscore (*p* = 0.0004) at week 6. However, the 25 U group was not reported to show a significant difference from placebo.

## 4. AAN Practice Guidelines

AAN practice guidelines for the botulinum neurotoxin treatment of dystonia were first published in 2008, at which time recommendations were made for blepharospasm, cervical dystonia, laryngeal dystonia, and focal upper limb dystonia [18]. Data were deemed inadequate to make recommendations regarding focal lower limb dystonia. Updated AAN practice guidelines for the botulinum neurotoxin treatment of blepharospasm and cervical dystonia were published in 2016 on the basis of new research [19]. Laryngeal dystonia and focal upper limb dystonia were not rereviewed in the 2016 update.

The updated 2016 AAN practice guidelines for the treatment of cervical dystonia and blepharospasm are presented in Table 3. The task force evaluated evidence from randomized masked trials for efficacy but included evidence from nonrandomized trials to assess safety and long-term outcomes. The recommendations were evidence-based but were not restricted to regulatory-approved indications. Toxins were considered individually, not as a class. The task force identified 23 studies for cervical dystonia and 23 studies for blepharospasm which met quality inclusion criteria for review.

Of the articles analyzed for cervical dystonia, seven class I and four class II randomized masked trials were highlighted. All four toxins had at least one class I placebo-controlled trial. OnaBoNT-A had several class I or class II head-to-head trials against AboBoNT-A and RimaBoNT-B. Based on these trials, levels of evidence were assigned to each of the toxin formulations for the treatment of cervical dystonia. Level A requires at least two consistent class I studies. Level B requires one class I study or at least two consistent class II studies. Level C requires one class II study or at least two consistent class III studies. Level U indicates inadequate data were available. The task force concluded AboBoNT-A and RimaBoNT-B had level A recommendations, signifying the intervention should be offered for the treatment of cervical dystonia. OnaBoNT-A and IncoBoNT-A were assigned level B recommendation, signifying these toxins should be considered for administration to patients with cervical dystonia. The task force additionally concluded that OnaBoNT-A and RimaBoNT-B were equivalent in efficacy for CD and that OnaBoNT-A and AboBoNT-A were probably equivalent in efficacy. Despite differing levels of evidence, the authors noted that all four formulations are commonly used in practice [19].

In 2008, the task force for the original AAN guidelines for BoNT treatment of blepharospasm identified two class II placebo-controlled trials of OnaBoNT-A [15,20] in addition to one class II [21] and one class III [22] head-to-head trial of OnaBoNT-A against AboBoNT-A. The single class I study identified compared OnaBoNT-A and IncoBoNT-A [23]. In the 2016 guideline review, additional trials had been completed: one class I placebo-controlled trial [16] for IncoBoNT-A, one class II placebo-controlled trial for AboBoNT-A [24], and one class I [25] and one class II [26] head to head trial comparing OnaBoNT-A and IncoBoNT-A. Four class IV observational studies reported sustained benefits with long term use of all three BoNT-A toxins for at least 5 to 15 years [27,28,29,30]. As none of the toxins had two class I trials evaluating efficacy for treatment of blepharospasm, none received a level A recommendation for this indication. Both OnaBoNT-A and IncoBoNT-A received a level B recommendation, suggesting they should be considered. AboBoNT-A received a level C recommendation suggesting it may be considered. RimaBoNT-B received no recommendation given insufficient evidence. The task force noted that most movement disorder specialists consider BoNT to be first line in the treatment of blepharospasm and concluded that all three type A toxins appear to have similar efficacy with continued benefit over time.

While the 2016 guidelines did not review BoNT treatment for other forms of dystonia, the 2008 guidelines reviewed pertinent literature for focal limb dystonia and laryngeal dystonia [18,19]. The reviewing task force noted that for focal limb dystonia, most studies had been conducted for only upper extremity dystonia. A level B recommendation was made for use of botulinum neurotoxin in focal upper extremity dystonia on the basis of one class I [31] and three class II studies [32,33,34]. No recommendation was made for the botulinum neurotoxin treatment of isolated lower limb dystonia given inadequate data.

A single class I study for adductor spasmodic dysphonia [35] was identified by the task force in 2008, leading to a level B recommendation of BoNT in its treatment. It concluded there was insufficient evidence to evaluate the use of BoNT in abductor spasmodic dysphonia leading to a level U designation for this condition [18].

## 5. European Federation of the Neurological Societies Practice Guidelines

The most recent European Federation of the Neurological Societies (EFNS) guidelines on diagnosis and treatment of primary dystonia were published in 2011 and include recommendations and good practice points regarding BoNT treatment of focal dystonia [36]. The guidelines state that level A evidence exists for the use of BoNT-A (or BoNT-B in the case of BoNT-A resistance) as first-line treatment for primary cranial (excluding oromandibular) or cervical dystonia. They state that there is level A evidence for BoNT-A as effective in writer’s cramp, but that BoNT-A is only possibly effective in other types of upper limb dystonia. BoNT-A is assessed to be probably effective for adductor-type laryngeal dystonia, but with insufficient evidence to support efficacy in abductor-type laryngeal dystonia. Additional recommendations and good practice points are made regarding use of EMG or ultrasound and other topics (Table 4).

## 6. Off-Label Uses of Botulinum Neurotoxin Injections for Dystonia

While the only forms of dystonia for which there are FDA-approved indications for botulinum toxin are blepharospasm and cervical dystonia, BoNT is routinely administered for other forms of dystonia “off-label” including, but not limited to, spasmodic dysphonia, oromandibular dystonia, truncal dystonia, limb dystonia, and tardive dystonia [19,39,40]. For BoNT treatment in many forms of dystonia, the literature consists primarily of open-label studies and clinical series rather than controlled trials. Nonetheless, higher-level evidence is needed to better guide clinical care in many of these indications.

### 6.1. Limb Dystonia

Relatively few trials have been completed for limb dystonia, with the majority completed in the late 1990′s to mid-2000′s [31,32,33,34]. The only class I trial was a randomized, double-blind trial of forty patients with writer’s cramp receiving AboBoNT-A or placebo [31]. Seventy percent of the AboBoNT-A versus 31.6% of the placebo group reported beneficial effect and chose to continue treatment, which was the primary outcome measure. However, participants were subsequently followed for one year and only 51% continued BoNT with positive effects. In a smaller placebo-controlled, double-blind, crossover study, 17 patients with limb dystonia were found to have greater improvement in symptom control with BoNT compared to placebo injections in subjective patient reports only, with 82% vs. 7% reporting improvement [32]. In contrast, objective measures failed to show significant improvement in BoNT compared to placebo. Another study, a class II placebo-controlled, double-blind, crossover trial of 20 patients with writer’s cramp showed significant improvement in speed and accuracy of pen control in a task in which participants were asked to aim a pen at dots on a tablet screen [33]. However, only four subjects had improvement in writing after OnaBoNT-A in objective or subjective measurements. The authors further subdivided patients into those with and without marked joint deviation as a feature of their dystonia and concluded that those with observable wrist deviation appeared to have better symptom improvement with BoNT compared to those without. In another class II double-blind, placebo-controlled, crossover study of ten patients with focal hand dystonia, 80% had improved subjective rating and 60% had improved videotape rating with BoNT compared to placebo [34]. Collectively, these early investigations highlight difficulties with trial design for BoNT treatment of upper limb dystonia, including difficulty designing objective rating methods and scales in limb dystonia that adequately capture symptom improvement, and potentially differential response with variable clinical phenotypes (e.g., wrist deviation vs. clenching pattern in writer’s cramp).

In the years since these earlier studies, there has been a paucity of blinded trials in BoNT treatment of upper limb. However, a randomized, double-blind, placebo-controlled crossover study of IncoBoNT-A in participants with musician’s dystonia affecting one or both upper limbs is currently underway [41] in addition to a multicenter double-blind, placebo-controlled randomized trial of IncoBoNT-A in focal hand dystonia [42].

There have been no recent published controlled trials for use of BoNT in lower limb isolated dystonia, but there has been one double-blind, randomized trial for the treatment of painful dystonic toe flexion in Parkinson’s disease patients with IncoBoNT-A injections [43]. In this trial, 45 patients were injected with 100 units of IncoBoNT-A or placebo in the flexor digitorum longus or flexor digitorum brevis in two sessions three months apart. The primary endpoint was Clinical Global Impression of change six weeks after each injection. The IncoBoNT-A group significantly improved compared to the placebo group (*p* = 0.039) and no difference was found between groups receiving IncoBoNT-A in one muscle as compared to the other. The complexities of good trial design for BoNT treatment of limb dystonia are highlighted above, and the scarcity of large, well-controlled trials is notable. This should be a priority in future research in order to better guide clinical care.

### 6.2. Laryngeal Dystonia

The literature in BoNT treatment of laryngeal dystonia, including spasmodic dysphonia (SD), is marked by a notable scarcity of controlled trials. Despite the lack of clinical trial data, BoNT injections are regarded by many as first-line treatment for SD primarily due to the degree of benefit noted in open-label studies and lack of alternative treatment approaches [44,45]. One randomized, double-blind, parallel group study consisted of only 13 patients with adductor spasmodic dysphonia who received saline or BoNT injections. Those in the treatment group had significantly greater subjective and objective improvements in dystonia (*p* = 0.01). Adverse effects in the treatment group included excessive breathiness in two patients and mild bleeding in one patient. Other data regarding BoNT treatment of laryngeal dystonia primarily come from case series, including a 1300 patient longitudinal case series over 24 years [44]. Within this cohort, 82% of patients had adductor (ADSD) and 17% had abductor spasmodic dysphonia (ABSD). Treatment approaches were variable, including both unilateral and bilateral injections and variation in timing, frequency, and dosing of BoNT. Overall, the ADSD patients experienced an average duration of benefit of 15.1 weeks with most frequent side effects of breathy voice (25%) and transient coughing when drinking fluids (10%). The ABSD group experienced an average 10.5 weeks of benefit with most common side effects of mild transient dysphagia of solids (6%) and mild exertional wheezing (2%). Despite many years of clinical experience documented in open-label series, well-designed trials are still needed in order to offer a higher level of evidence for BoNT treatment of laryngeal dystonia, particularly in less common presentations such as ABSD.

### 6.3. Oromandibular Dystonia

Oromandibular dystonia (OMD) was not addressed in the 2008 or 2016 AAN guidelines and evidence available for its treatment with BoNT comes primarily from open-label observational studies and retrospective chart reviews [18,19]. Despite the lack of well-designed trial data, BoNT injections are considered to be the preferred treatment by many [45,46]. One double-blind, placebo-controlled study of BoNT in cranial-cervical dystonia included ten patients with combined CD and OMD [15]. Only a minority had improvement with OnaBoNT-A injections [47]. However, the low number of participants and combined phenotype of CD and OMD in this study limit conclusions about generalizability of these results to patients with OMD alone.

As with laryngeal dystonia, much available data come from open-label series, including one with 162 patients with OMD treated with OnaBoNT-A injections into the masseters and submentalis complex over a 10-year period [48]. Eighty-five of these patients had jaw closing OMD, 35 had jaw opening OMD, 39 had mixed OMD, and 3 had jaw deviation OMD. Treatment response was assessed by the Global Rating Scale (GRS) where 0 = no effect and 4 = marked improvement in severity and function. One point was subtracted for mild to moderate complications from the injection, and two points were subtracted for severe complications. One-hundred ten patients (67.9%) received a global score of three or higher. Of the patients with jaw closing dystonia, 68 patients (80%) improved by three points on the GRS. In contrast, of those with jaw opening dystonia, only 20 patients (57.1%) improved by at least three points. Fifty-one patients (31.5%) experienced adverse events, most frequently dysarthria, but only 11.1% of the 1213 treatment visits were complicated by an adverse event. A recent meta-analysis of BoNT in isolated OMD [49] included seven open-label and two retrospective studies with a total of 387 patients. A total of 49.8% of patients were found to have a favorable response to BoNT injections and 27.1% of participants experienced complications, predominantly dysphagia. Notably, none of the included studies were double-blind, randomized, controlled trials.

More recently, a pilot single-blind study was published evaluating AboBoNT-A dosing and efficacy in OMD [50]. In an initial dose-finding phase, three subjects were injected with AboBoNT-A into muscles chosen by the injector based on the pattern of dystonia, using prespecified fixed doses assigned to each of the muscles eligible for injection (anterior digastric, genioglossus, masseter, medial pterygoid and lateral pterygoid). As two of the three subjects experienced mild side effects even at these doses, no further dose escalation was performed as previously planned, and the low dose scheme was used for the next phase, a single injection session for exploration of safety and efficacy. Eighteen patients were enrolled in this phase, fifteen with idiopathic OMD and three with tardive OMD. Nine had jaw-opening, three had jaw-closing, and six had a combined jaw opening and closing pattern. Six had intermixed tongue protrusion. All received injections tailored to their presentation using fixed doses of 7.5 to 50 units AboBoNT-A in muscles selected from the same set allowed in phase 1. Efficacy measures, including the jaw/tongue portions of the Global Dystonia Severity Rating Scale (GDS) and Unified Dystonia Rating Scale (UDRS), showed significant improvement compared to baseline at 6 and 12 weeks in unblinded ratings (*p* < 0.001), but not in blinded video assessments. Measures of quality of life and speech in addition to Clinical Global Impression (CGI) improvement and severity were also significantly improved. Nine of the eighteen patients experienced mild to moderate side effects, most commonly dysphagia. However, a safety evaluation was conducted after the first ten injections and revealed that of five patients receiving injections for lingual dystonia, four had developed dysphagia. Subsequent dosing of the genioglossus muscle was decreased from 15 units to 7.5 units, after which no additional genioglossus injections resulted in dysphagia. This was a small pilot study without placebo control, so limited conclusions can be drawn from its results regarding efficacy. However, it offers valuable data regarding dosing to help guide future studies in AboBoNT-A in this condition. As with laryngeal dystonia, high-level evidence guiding BoNT treatment of OMD is limited and additional well-designed trials evaluating injection technique, dosing, and efficacy are needed.

### 6.4. Truncal Dystonia

There are scarce data on BoNT treatment of truncal dystonia. A small single-blinded study evaluated the efficacy of BoNT on truncal dystonia resulting in truncal extension and pain [51]. Five patients were injected with 150 to 300 units of OnaBoNT-A into the lumbar paravertebral muscles and were rated at baseline and at 2–4 weeks with a score evaluating truncal dystonia (from 0, no dystonia to 10, extreme arching and bending.) Blinded raters found that three patients had a mean improvement of 37% while two had no objective improvement. However, all patients reported less pain and increased range of motion, suggesting a need for additional investigation.

## 7. Other Toxins not FDA-Approved for Dystonia

There are currently a number of botulinum neurotoxin formulations not FDA-approved for the treatment of dystonia, including Prabotulinumtoxina A-xvfs, Daxibotulinumtoxin A (DaxiBoNT-A), Neu-botulinumtoxinA (NeuBoNT-A), Letibotulinumtoxin A, botulinum toxin E (rBoNT-E), Innotox, and QM-1114. Of these, DaxiBoNT-A, NeuBoNT-A, and rBoNT-E are in trials for use in dystonia. DaxiBoNT-A is a novel BoNT with a stabilizing peptide to promote longer-lasting effects. It has no animal-derived components or human albumin, similar to IncoBoNT-A, and does not require refrigeration with a shelf-life of two years [52]. This toxin was shown to have a mean response duration of 25 weeks in CD in a phase II study [53], and a phase III multicenter study of its use in CD is currently underway [54].

NeuBoNT-A, alternatively called Korean botulinum toxin A, Meditoxin, or Neuronox, is currently mostly used in Asian and Latin American countries. Two randomized trials compared NeuBoNT-A to OnaBoNT-A in blepharospasm and showed similar efficacy at week six [55,56]. In cervical dystonia, 20 patients were enrolled in a prospective, open-label, single-arm study of NeuBoNT-A which showed significant improvement at six weeks [57]. A head-to-head double-blinded, randomized controlled trial underway in Thailand is evaluating AboBoNT-A versus NeuBoNT-A for the treatment of cervical dystonia and is expected to be completed in October 2020 [58].

rBoNT-E is the first recombinant serotype to be studied in clinical trials. While serotype A binds to all three isoforms of synaptic vesical protein 2 (SV2A, SV2B, and SV2C), rBoNT-E binds only to glycosylated forms of SV2A and SV2B. In a phase 1 double-blind, placebo-controlled trial, 65 healthy males were injected with rBoNT-E, AboBoNT-A, or placebo into the extensor digitorum brevis muscle under ultrasound guidance. rBoNT-E was well-tolerated though 52% reported a mild or moderate treatment associated adverse event. In the rBoNT-E group, time to onset ranged from 1–2 days compared to 1–7 days for the AboBoNT-A group. The maximum effect for the rBoNT-E group was 1 week and the effect lasted from 10 to 50 days depending on the dose injected [59]. This shorter acting toxin may be useful given the rapid onset of action and shortened duration of effects in novel clinical applications and approaches to dystonia management.

## 8. Conclusions

This paper has reviewed the current FDA approved BoNT formulations for various dystonia indications as well as the established guidelines by both the AAN and EFNS. The field is expanding, and in practice BoNT is commonly used off-label in many other types of dystonia. While considered first-line therapy in many forms of dystonia, high-level data are lacking for many off-label uses, and well-designed trials are needed in order to better guide clinical treatment of dystonia with BoNT. With several new BoNT formulation now in development and being tested in dystonia, the therapeutic options are likely to grow in the near future.

## Figures and Tables

**Table 1:** Federal Drug Administration (FDA)-approved botulinum toxins for use in cervical dystonia.

	OnaBoNT-A	RimaBoNT-B	AboBoNT-A	IncoBoNT-A
**Year of FDA Approval**	1999	2000	2009	2010
**Pivotal Trial**	2012 [1]	1997 [2] 1999 [3]	2005 [4] 2010 [5]	2011 [6]
**Max FDA Recommended Total Dose**	400 U	10,000 U	1000 U	400 U

**Table 2:** Current FDA approved BoNT formulations for dystonia.

Cervical Dystonia	Blepharospasm
OnaBoNT-A	OnaBoNT-A
IncoBoNT-A	IncoBoNT-A
AboBoNT-A	
RimaBoNT-B	

**Table 3:** American Academy of Neurology (AAN) clinical practice guidelines for botulinum neurotoxin treatment of dystonia.

	Cervical Dystonia	Blepharospasm
**Level A**	AboBoNT-A RimaBoNT-B	
**Level B**	IncoBoNT-A OnaBoNT-A	IncoBoNT-A OnaBoNT-A
**Level C**		AboBoNT-A
**Level U**		RimaBoNT-B

Adapted from Simpson et al. 2016 [19]. Level A: intervention should be offered. Level B: intervention should be considered. Level C: intervention may be considered. Level U: insufficient evidence to support or refute effectiveness of intervention.

**Table 4:** European Federation of the Neurological Societies (EFNS) practice guidelines for botulinum neurotoxin treatment of dystonia.

Practice Points and Recommendations
Level A: BoNT/A (or type B if there is resistance to type A) can be regarded as first-line treatment for primary cranial (excluding oromandibular) or cervical dystonia [37,38].
Good practice point: BoNT/A is effective for writer’s cramp [31] (level A) and is possibly effective in other types of upper limb dystonia, but controlled dose adjustments are needed because of frequent muscle weakness.
Good practice point: BoNT/A is probably effective for adductor-type laryngeal dystonia, but there is insufficient evidence to support efficacy in abductor-type laryngeal dystonia and in muscular tension dysphonia.
Good practice point: BoNT injections are safe and efficacious when repeated treatments are performed over many years (good practice point), but doctors and patients should be aware that excessive cumulative doses may be dangerous, particularly in children.
Good practice point: BoNT injections can be performed by direct inspection; EMG- or ultrasound-assisted targeting may improve clinical outcome.
Good practice point: BoNT should not be used in patients affected by a disorder of neuromuscular transmission or in presence of local infection at the injection site. The recommended dosage should not be exceeded [36].
